# Controlled liquid-liquid phase separation via the simulation-guided, targeted engineering of the RNA-binding protein PARCL

**DOI:** 10.1016/j.isci.2025.112852

**Published:** 2025-06-11

**Authors:** Ruth Veevers, Steffen Ostendorp, Anna Ostendorp, Julia Kehr, Richard J. Morris

**Affiliations:** 1 Computational and Systems Biology, John Innes Centre, Norwich, UK; 2 Universität Hamburg, Department of Biology, Institute of Plant Science and Microbiology, Hamburg, Germany

**Keywords:** Molecular dynamics, Biomolecules, Protein, Bioengineering, Biophysics, Computational bioinformatics

## Abstract

The Phloem-Associated RNA-Chaperone-Like (PARCL) protein is a plant-specific RNA-binding protein (RBP) that is highly abundant in the phloem. PARCL has been observed to form large biomolecular condensates that move within the phloem stream, potentially being involved in RNA transport. Here, we present results on unraveling drivers for PARCL’s phase separation. We used coarse-grained molecular dynamics simulations to compute a residue interaction map that identifies candidate residues involved in phase separation. Subsequent simulations with mutations of candidate residues resulted in disrupted condensation, supporting their involvement in phase separation. We performed *in vitro* and *in vivo* experiments to validate these predictions. To investigate the RNA-binding of PARCL, we added microRNA to the simulations and identified a short region of PARCL that consistently made contact with the miRNA in agreement with bioinformatics predictions and experiments. We discuss the implications of our findings in terms of model-guided engineering of biomolecular condensates.

## Highlights

• Simulations of Phloem-Associated RNA-Chaperone-Like protein show phase separation
• Contact analysis identifies key residues that drive phase separation
• Simulations capture the influence of microRNA on PARCL phase separation
• Mutations separately affect phase separation and condensates’ miRNA recruitment

## Graphical abstract

**Figure:**
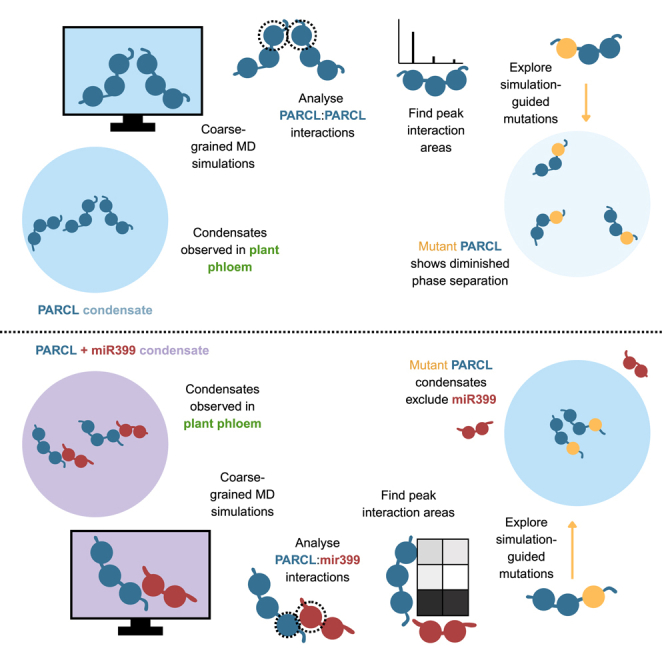

## Introduction

Biomolecular condensates are membraneless compartments, such as stress granules and nucleoli, that perform key biological functions.^1^^,^^2^^,^^3^ These condensates are often formed by the process of liquid-liquid phase separation (LLPS), wherein biomolecules become partitioned from their surrounding liquid environment into liquid droplets with their own composition and dynamics.^4^^,^^5^ From a thermodynamic viewpoint, we would expect condensate formation to be sensitive to macroscopic factors, such as temperature, pressure, and concentration, and these dependencies have been experimentally demonstrated, leading to the determination of phase diagrams for different specific proteins.^5^^,^^6^^,^^7^ From a molecular viewpoint, we would expect the strength and frequency of intermolecular interactions to be important and indeed relationships between molecular composition and phase separation have been discovered.^8^^,^^9^ For instance, intrinsic disorder and the presence of a prion-like domain (PLD) are common to phase separating proteins.^10^^,^^11^ Amino acid properties, such as hydrodynamic size, propensity for beta-turns, and sequence hydrophobicity, also influence condensate formation, and the amino acid distribution differs significantly between disordered proteins that are prone to phase separation and those that are not.^12^ Multivalent interactions drive condensate formation, and the types and patterning of proteins’ amino acids can suggest which interactions are possible for a protein. The contributions of electrostatic interactions are dictated by charged residues and how they are distributed within the protein sequence.^13^ Aromatic residues are a strong determinant of phase separation behavior^14^ due to their contribution to π-π and cation-π interactions.^15^

Key insights into phase separation have been gained from computational studies, with approaches ranging from 1D sequence analyses, such as the method of residue-counting proposed by Wang et al.*,*^11^ to spatiotemporal analyses, such as molecular dynamics (MD) simulations that calculate the trajectories of individual atoms.^16^ While recent advances are rapidly expanding the range of applicability,^17^^,^^18^ studying large systems over long timescales using all-atom MD simulations can quickly become computationally prohibitive. To address this limitation, a common approximation is to build coarse-grained models, where groups of atoms are represented by a single particle or “bead”.^14^^,^^19^^,^^20^^,^^21^^,^^22^^,^^23^ The bead and interaction parameters are determined from experimental data and by the outcomes of smaller all-atom simulations. Dignon et al.^24^ proposed two coarse-grained, one-bead-per-residue models in a slab simulation framework: the KH model is based on the Miyazawa-Jernigen potential for inter-residue attractions; the HPS model is based on a hydrophobicity scale.^25^ This level of approximation allows for residue-specific effects to be taken into account. Schuster et al.^26^ used coarse-grained MD to simulate the disordered domain of the LAF-1 protein, finding that a short, conserved, hydrophobic region was driving phase separation. To assess the relative effects of electrostatic, hydrophobic, π-π and cation-π interactions, Das et al. ^44^ produced simulations using the models proposed in Dignon et al.^24^, both as published and tailored to include cation-π interactions. They simulated the IDR of the Ddx4 protein and three variants with less propensity to phase separate, identifying areas of agreement and disagreement between simulations produced by the HPS model and phase separation behavior ascertained experimentally. Regy et al.^27^ improved the HPS model’s agreement with experimental observations by replacing the Kapcha-Rossky hydrophobicity scale with that of Urry et al*.*^28^

Based on advances towards developing a molecular grammar that explains phase separation, Kilgore and Young^29^ anticipated a complete grammar that would clarify the relationships between amino acid sequence features and phase separation propensity. Molecules could thus be designed that could be specifically recruited into a condensate, or that could modulate the phase behavior or material properties in a system. Many studies have attempted to find mutations that would change an existing protein’s LLPS propensities.^30^^,^^31^ These mutations were proposed using various methods: predictive screening of exhaustive random mutations^32^; patient-derived mutations^33^; or *in silico* homology modeling.^34^ Coarse-grained MD simulations have been used to find short regions to delete based on contact probability,^26^ to guide the progress of genetic algorithms,^35^ and to form the basis of shorter or smaller all-atom simulations.^16^

**Figure 1:**
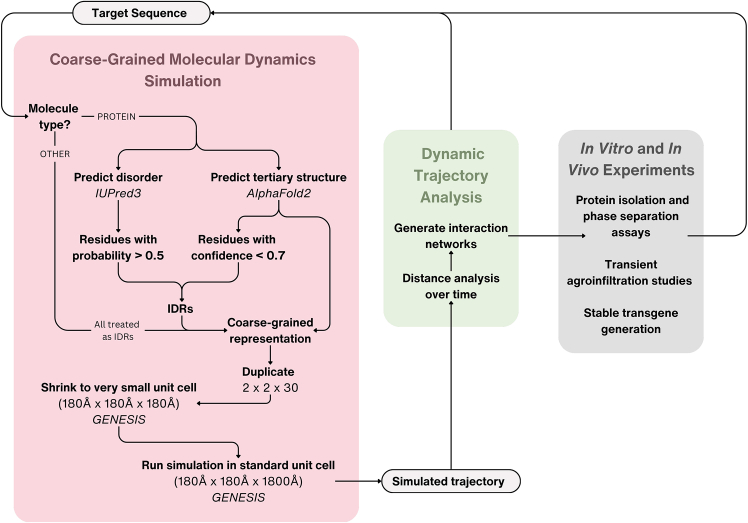
Representation of the framework used to generate molecular dynamics simulations from a sequence, analyze the resulting trajectories, and propose mutations to disrupt phase separation

In this work, we use the coarse-grained HPS-Urry model^27^ in the GENESIS MD software^36^ to simulate the phase separating *Arabidopsis thaliana* Phloem-Associated RNA-Chaperone-Like (PARCL) protein (Figure 1). Atomistic simulations have been used to make confident assessments of the specific interactions driving LLPS.^16^^,^^26^ However, this is a computationally expensive process, whereas the speed and ease of the coarse-grained model offers the possibility of high-throughput data generation that may provide sufficient insights to guide targeted engineering. The target protein, PARCL, is an RNA-binding protein (RBP) recently characterized^37^ as a phloem-abundant, intrinsically disordered protein that exhibits chaperone activity and contains a PLD. PARCL proteins form large condensates observed *in vivo and in vitro*, *highlighted with the addition of using fluorescent eYFP domains.* The presence and choice of fluorescent tags can affect LLPS.^38^^,^^39^ Therefore, we investigated the effects of eYFP on PARCL’s phase separation behavior. We score individual residues using a radius-based contact distance estimation to predict which interactions may drive phase separation, and validate these findings experimentally by mutation of the most highly scoring residues. We demonstrate that inducing the mutations predicted by the simulations can indeed prevent the formation of condensate droplets *in vitro* and in plant leaves *in vivo*. Adding microRNA to the system using the parameters defined by Regy et al.^27^ allows us to recapitulate PARCL’s experimentally determined RNA-binding behavior both in the binding location and in the observed effect on condensates. We therefore demonstrate that residue-level approximations are sufficient to guide targeted engineering that influences phase separation and RNA binding of PARCL and, potentially, other proteins.

## Results

### Phase separation of eYFP-PARCL can be attributed to PARCL

**Figure 2:**
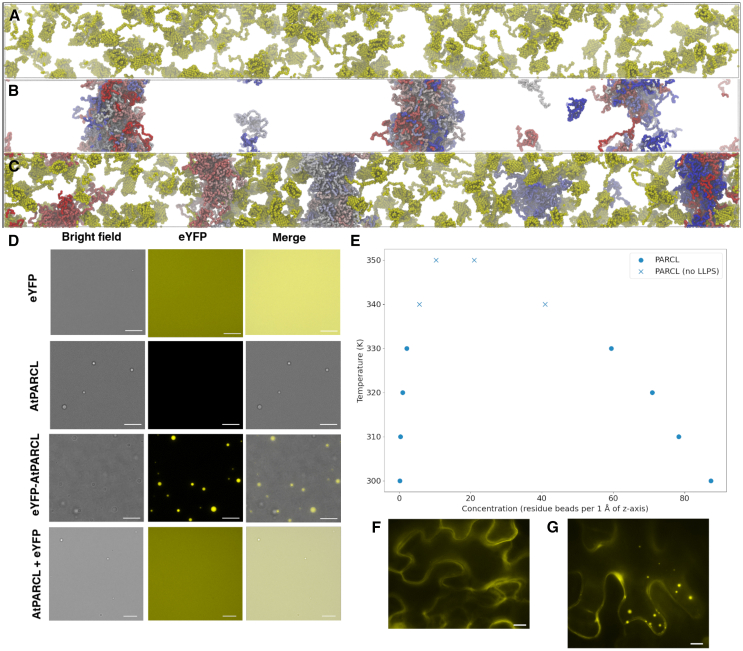
Condensation of eYFP-PARCL is driven by the PARCL domain rather than the eYFP domain (A) Slab simulation of 120 structured eYFP proteins after 100 nanoseconds of coarse-grained MD simulation. No phase separation can be observed. Rendered in VMD using the Tachyon ray-tracer. (B) Slab simulation of 120 disordered PARCL proteins after 100 nanoseconds of coarse-grained MD simulation. The majority of the PARCL proteins are located in three dense “droplets”, with some isolated molecules and smaller clusters remaining in the dilute phase. Rendered in VMD using the Tachyon ray-tracer. (C) Slab simulation of 120 free eYFP proteins (yellow) and 120 wild-type PARCL proteins (colors along the blue-white-red spectrum), using default interaction and energy parameters. The PARCL proteins are clustered in 5 “droplets” which do not recruit the eYFP molecules; compared to the PARCL proteins alone in (B), there are more, smaller droplets shown in (C), and fewer isolated PARCL proteins. Rendered in VMD using the Tachyon ray-tracer. (D) Experimental validation of simulation results from A to C. Proteins were tested for their condensation behavior after addition of 10% PEG3350: free eYFP alone; free AtPARCL; eYFP fused to AtPARCL; an equimolar mixture of free eYFP and free AtPARCL. Whereas free eYFP did not show any phase separation, free AtPARCL and a mixture of free eYFP and AtPARCL did and were in good agreement with the obtained simulation results. All proteins were measured at 10 μM concentration in 1x condensation buffer and 10% PEG3350 with the same exposure time (17 ms). Pictures were taken using bright field and YFP fluorescence. Scale bar: 10 μm. (E) Phase diagram showing temperature/concentration relationship across temperatures. Density plotted as the mean of the final 100 ns of a 300 ns simulation. At temperatures where LLPS was deemed not to have occurred, density had not converged by this time. (F) *In vivo* observation of free eYFP in agroinfiltrated tobacco leaves. The transiently expressed free eYFP did not show any condensates. Scale bar: 10 μm. (G) *In vivo* observation of PARCL condensates in agroinfiltrated tobacco leaves. Wild-type PARCL N-terminally fused to eYFP was transiently expressed in tobacco leaves. EYFP-PARCL showed several cytosolic condensates throughout the cells, whereas free eYFP (F) did not show any condensates. Scale bar: 10 μm.

We previously reported that eYFP-PARCL undergoes LLPS.^37^ The presence of eYFP might inadvertently change the overall phase separation propensity of the fusion construct compared to the untagged protein. Despite using identical molar concentrations in all assays and simulations, eYFP may increase potential crowding effects as long as the overall reaction volume and conditions are kept constant, potentially influencing condensate size, number, or fluidity when compared to untagged proteins. To investigate the impact of eYFP on phase separation, we conducted simulations using eYFP and PARCL alone. The FuzDrop^40^ LLPS predictor does not predict that eYFP undergoes phase separation; its score (pLLPS) of 0.4443 falls below the 0.5 threshold. In line with this, the MD simulations of eYFP show an approximately uniform distribution of molecules (Figure 2A). We next carried out MD simulations for PARCL (pLLPS = 0.9969) without the eYFP tag. These simulations exhibit clear phase separation behavior (Figure 2B) with PARCL forming three main droplets with some individual proteins remaining in the bulk solvent. The phase diagram in Figure 2E shows the estimated concentration/temperature dependence of PARCL LLPS. We next simulated the PARCL and eYFP proteins as separate molecules in the same system. Figure 2C shows that PARCL proteins formed condensed droplets while the eYFP remained in the bulk solvent. We also ran simulations for eYFP-PARCL, Figure S1. While eYFP-PARCL reproducibly forms liquid clusters, slab simulations failed to robustly maintain localized concentrations, thus rendering these results inconclusive. We speculate that this is likely due to different force fields in the simulations for describing large, structured domains and disordered regions.

*In vitro* experiments confirmed that eYFP alone does not form condensates, but that eYFP-PARCL does *in vitro* and *in vivo* (Figures 2D–2F and 2G). Consistent with the simulations (Figure 2C), added free eYFP did not interfere with PARCL condensate formation and was excluded from condensates (Figure 2D).

From these results, we conclude that coarse-grained MD simulations can successfully reproduce condensate formation of the PARCL protein (with and without eYFP) and that PARCL alone is sufficient to induce phase separation, in line with *in vitro* studies (Figure 2D).

### Contact dynamics from MD simulations can be used to predict key condensate-inducing residues

**Figure 3:**
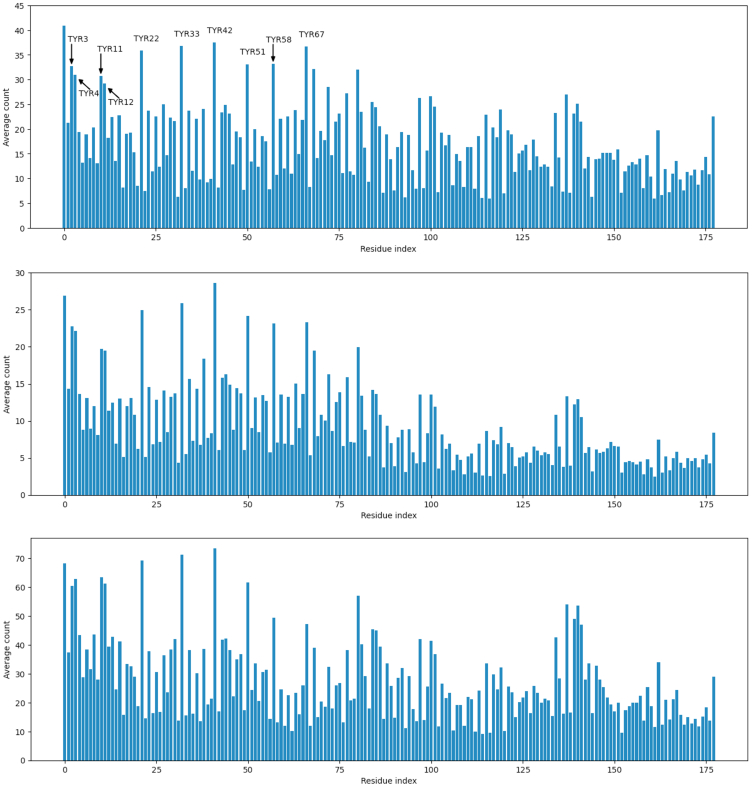
Potential contact frequency between residues determines key drivers for phase separation Mean potential contacts per frame for the 120 copies of each residue of the wild-type PARCL protein during 1000 frames of coarse-grained MD simulation, lasting 100 nanoseconds. Simulations produced using three models for phase separation. Top: HPS-Urry^41^ (last 100 ns from 400 ns simulation; error bars shown in Figure S5); Middle: model proposed by Tesei et al.^42^; Bottom: HPS-FB.^43^ Two residues from different PARCL molecules are considered in potential contact if the distance between the coordinates of their representative beads is less than the sum of their radii. While potential contacts were spread across the whole PARCL sequence, there were more noticeable peaks in its N-terminal half. Many of the peaks most often falling within contact distance are tyrosine residues, which are labeled by residue index.

Having gained confidence in the ability to reproduce experimental observations of PARCL condensate formation *in silico*, we sought to determine the driving factors for this phenomenon. Given that macromolecule:macromolecule interactions and multi-valency are known to be important for phase separation, we reasoned that those residues that come into contact with other molecules might play a role in PARCL condensate formation. To estimate contact frequencies, we used the molecular dynamics trajectories to identify pairs of residues that pass within contact distance, as calculated from the radii of each amino acid. Analyzing these potential interactions between residues of different PARCL molecules allowed us to determine which parts of the protein are contributing to the phase separation behavior observed in the simulation. Figure 3 shows how many intermolecular contacts each of the PARCL residues make, averaged across the simulation. As individual models can differ in residue-level behavior, we repeated this process using two other models available within GENESIS, finding that the patterns observed were maintained. Aromatic residues, particularly tyrosine, have been shown to participate in interactions that promote LLPS.^14^^,^^44^ In agreement with this, the simulations showed that, apart from the leading methionine, PARCL’s 10 tyrosines make the most intermolecular contacts. The first four tyrosines are located in pairs at the start of the sequence, and the remaining six appear throughout the protein’s PLD. The four paired residues were the least-interacting tyrosines. To confirm that the condensed phase was liquid-like, we compared the contacts between frames, observing how often contacts are made and broken. In line with the nature of intermolecular interactions expected of liquids, we found that the contacts were transient: in the HPS-Urry PARCL simulation, 99.7% of the residue pairs that came within contact distance in a frame of a trajectory had separated by the next frame, 100 picoseconds later. This supports our visual assessment that the droplets were highly dynamic and fluid-like. Our simulations of eYFP-PARCL did not accurately reflect experimental findings (Figure S1) and resulted in many smaller liquid clusters instead of the slab-like droplets seen in simulations of the untagged PARCL protein (Figure S2). However, local analysis of contacts revealed similar patterns (Figure S3).

The key interactions in the protein can be visualized in a network (Figure S4) where nodes represent residues and edges indicate contacts between the residues. Many of the residues encountered one another across the entire trajectory (for clarity Figure S4 shows only interactions that occurred at least 350 times within the 1,000 frames of the final 100 ns of the full 400 ns simulation). The aromatic residues of the PLD formed a large, well-connected cluster in the network through frequent interactions with one another as well as contacts with other residues, particularly leucine residues adjacent to the PLD. The residues at each end of the sequence were in the subset of high-frequency contact residues, suggesting a potential role in phase separation. The initial methionine residue was included in the PLD cluster, and the final aspartic acid made frequent contact with several residues in a short region toward the C terminal that were not highly contacted individually.

To summarize, the analysis of intermolecular residue contact frequencies can be used to pinpoint potential key residues in the protein.

### Targeted engineering of PARCL validates the predicted phase separation behavior

**Figure 4:**
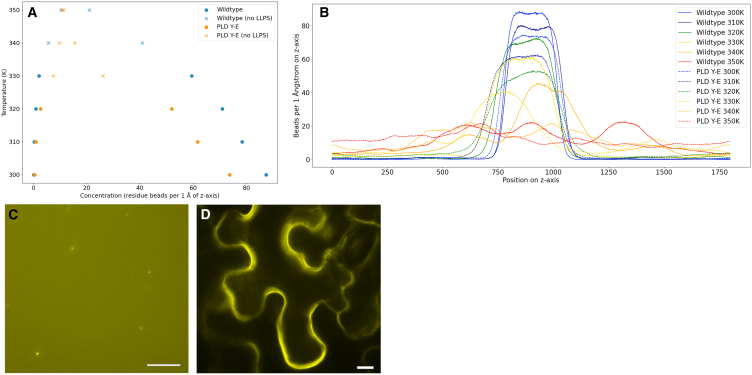
Mutating residues with high estimated contact frequency results in diminished phase separation The 6 tyrosine residues that were most often potentially contacted in the simulation of the wild-type (at indices 22, 33, 42, 51, 59, and 67) have been replaced with glutamic acid. (A) Phase diagrams for wildtype and mutant PARCL proteins. Concentration represented as mean. (B) The density plots underlying the phase diagram (A) plot the mean of the densities measured in the final 100 ns of 300 ns simulation. (C) *In vitro* condensation of PARCL^PLD Y−E^ mutants. 10 μM protein was used and condensation was induced by adding 10% PEG3350. Single aggregates or protein precipitate was visible upon addition of PEG. These did not show typical spherical appearance under higher magnification. Scale bar: 10 μm. (D) EYFP-PARCL^PLD Y−E^*in vivo* condensation in agroinfiltrated tobacco leaves. In line with simulations and *in vitro* assays, PARCL^PLD Y−E^ was unable to form condensates in cells. Scale bar: 10 μm.

Having shown that the simulations can capture PARCL’s biocondensate formation, we proceeded to evaluate whether the simulations could be used for rational, targeted perturbations of LLPS. Using the simulations’ contact frequencies as a guide, we set up simulations with an *in silico* PARCL mutant that lacked the residues that we identified as driving the phase separation behavior. The 6 tyrosine residues of the PLD were replaced with glutamic acid (PARCL^PLD Y−E^), since phosphorylation studies on wild-type PARCL highlighted that tyrosine phosphorylation at these sites can prevent condensation.^37^ Figure 4B shows the effect of these mutations on simulations at different temperatures. To test these predictions, we tested experimentally if PARCL^PLD Y−E^ proteins can form condensates *in vitro* and *in vivo* in transiently overexpressing tobacco leaves.

Comparing the changes in phase separation *in vitro* and *in vivo*, Figures 6C and 6D, reveals that the *in vitro* results of using a PARCL mutant with 6 tyrosine residues in the PLD mutated to glutamic acid are in line with the predictions. In the experiments we included crowding agents to achieve the required concentration for *in vitro* phase separation. In the MD simulations, we did not mimic the exact concentration of PARCL and PEG, instead we used a high concentration of PARCL molecules to account for the crowding effect of PEG.

To summarize, we have shown the utility of intermolecular contact maps by making predictions for phase separation behavior that we could reproduce both *in silico*, *in vitro and in vivo*. This methodology may be of general applicability for predicting target residues for experimental perturbation.

### Alternate tyrosine mutations show importance of residue selection

The PARCL^PLD Y−E^ mutant was created by replacing the residues (tyrosines) that made the most frequent contacts in simulations of PARCL^WT^. To evaluate other possible sets of mutations, we varied the tyrosines chosen for mutation. From an initial set of wild-type PARCL coordinate and input files, we programmatically replaced various combinations of tyrosines with glutamic acid for MD simulation. We evaluated single and multiple mutations. Not all possible combinations of residues were explored for each set size due to the computational expense of evaluating all of the hundreds of possible sets. Each simulation was run for 300 nanoseconds.

**Figure 5:**
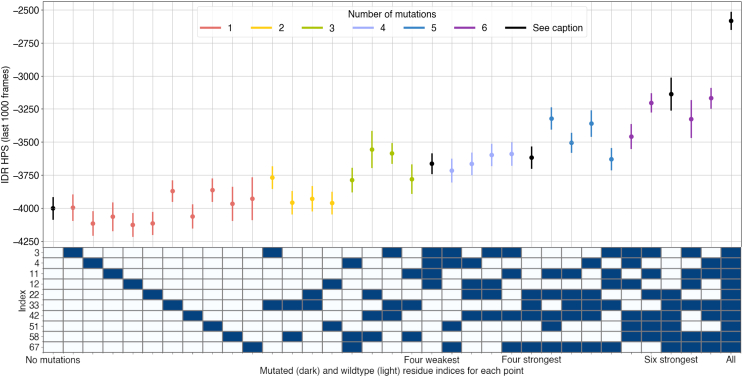
Fewer tyrosines in PARCL result in less pronounced LLPS However, choice of specific residues can be highly influential, which the model is able to reflect in these simulations. Points show mean IDR HPS energy contribution reported by the GENESIS simulation log files between frames 2,000 and 3,000 of the slab simulation (100 ns) trajectories with various mutations; lines show standard deviation. The PARCL^PLD Y−E^ mutant yielded the second-highest disruption, second only to the mutant in which all were replaced. In some cases the result is predictable from contact distance analysis of the wild-type simulation, but in others the apparent influence of residues is unexpected, indicating that the phase separation propensity of a sequence is not simply a result of a linear combination of individual residues’ contribution. The chart beneath the point plot indicates which residues are mutated. There is a row for each tyrosine in the wildtype PARCL sequence and a column for each mutation simulation (corresponding to the point directly above the column in the point plot). Blue cells indicate that the row tyrosine is mutated in the column simulation. Correlation between IDR HPS energy and phase diagrams shown in Figure S6.

We summarized the LLPS behavior for each mutant as a single metric for ease of comparison between simulations by using the IDR HPS energy reported by GENESIS for each frame of the simulation. This is the total interaction energy of the phase separation model.^41^ Fluctuation around a negative number indicates the formation of a condensate, whereas numbers closer to zero indicate a lack of LLPS. Figure S4 shows how this energy value relates to dense phase concentration across temperatures. We average across the final 50 frames of each simulation. Figure 5 shows the mean IDR HPS energy in this period for each of the MD simulations. The number of mutated residues appeared to be approximately negatively correlated with the HPS energy, but the choice of which residues to mutate can have a large effect.

**Figure 6:**
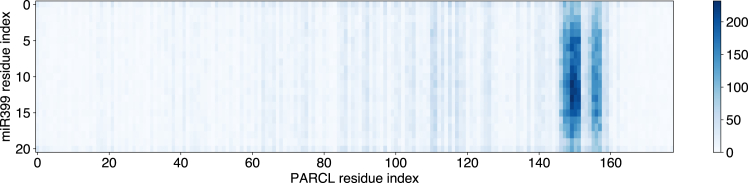
Potential contacts between PARCL and miRNA are mostly limited to a short, K-rich region toward the C-terminus The heatmap shows the mean number and location of potential contacts between residues in PARCL and in miR399 per frame of a coarse-grained MD simulation. Potential contacts are counted when a residue from a PARCL protein and a residue from miR399 are closer together than the sum of their standard radii. Potential contacts occur across the miR399 sequence, with a small preference for the central nucleotides. The PARCL proteins’ potential points of contact with the miRNA are largely limited to a short region toward the C-terminus rich in arginine, histidine, and lysine residues.

PARCL’s well-spaced tyrosine residues offer a long stretch of sequence that appears to be favorable for the multivalent interactions characteristic to phase separation. Our simulations show that these residues are promiscuous in regard to partner residues, interacting with one another and with other residues, so individual mutations of the tyrosine residues are not enough to prevent phase separation. MD simulations like these can capture the effects of fine-grained mutation combinatorics.

### Simulations of miRNA-PARCL interactions align with experiments in both the RNA interaction site and phase separation behavior

PARCL is known to bind miRNAs.^37^ To evaluate whether miRNAs have an impact on condensate formation, we added miR399 to the simulations.

### Simulations indicate a possible binding site containing most potential contacts between PARCL and miR399

When miR399 was included in the simulations, PARCL condensate formation occurred much as before, where the tyrosines in the PLD were the most frequently interacting residues in PARCL-PARCL contacts. PARCL-miR399 interactions, as shown in Figure 6, rarely occurred in PARCL’s PLD, instead being heavily focused on a short region toward the C-terminus. The high-contact region in Figure 5 covers the arginine, histidine and lysines around residue index 150. These residues are not themselves interaction hubs in the PARCL-only simulations but, as shown in Figure 3, they are each frequently contacted by the final aspartic acid. This K-rich region was indicated by Ostendorp et al.^37^ as a site for the binding of nucleic acids.

### Serine mutation in C-terminal domain has little effect on condensation

**Figure 7:**
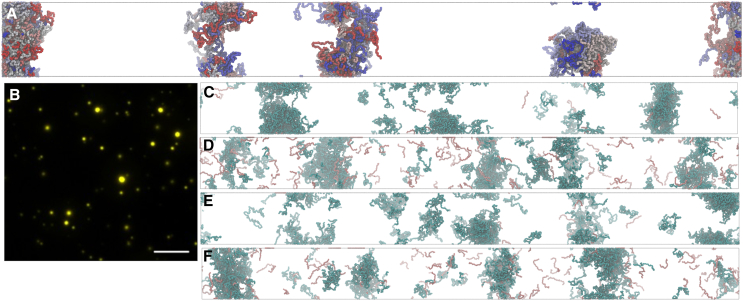
Mutations that reduce RNA binding do not substantially affect LLPS Additionally, phase separation of the wild-type and PARCL^C-term S−E^ mutant still occurs in the presence of miRNA, but simulations suggest smaller droplets. Serine residues near the C-terminus (at indices 169, 171, 172, 173, 175, and 177) have been replaced in the mutant. (A) Rendering of the final frame of the 100 nanosecond coarse-grained slab simulations performed on the mutated PARCL protein. All protein molecules are contained within four dense droplets, indicating phase separation as in the simulations of wild-type PARCL. (B) *In vitro* condensation of PARCL ^C−term S−E^ mutant. 10 μM protein was used and condensation was induced by adding 10% PEG3350. Scale bar: 10 μm. Small droplets are clearly visible. (C) Rendering of the final frame of the 100 nanosecond coarse-grained slab simulations performed on 120 mutated PARCL proteins (cyan) and 12 miR399 (red) present. The miR399 molecules were primarily in and around the PARCL droplets, and the droplets appeared smaller, with more PARCL proteins that are isolated or in small clusters with miRNA. (D) Rendering of the final frame of the 100 nanosecond coarse-grained slab simulations performed on 120 mutated PARCL^C-term S−E^ proteins (cyan) and 120 miR399 (red) present. There are miRNAs within and at the interface of the condensates, but also isolated throughout the bulk solvent, which could suggest a limit to the amount of miR399 that can be recruited by droplets of these sizes. (E) Rendering of the final frame of the 100 nanosecond coarse-grained slab simulations performed on 120 PARCL^WT^ proteins (cyan) and 12 miR399 (red) present. As in (C), the miR399 molecules are primarily in and around the PARCL droplets. The droplets are smaller, more abundant, and less well-defined than simulations without miRNA. (F) Rendering of the final frame of the 100 nanosecond coarse-grained slab simulations performed on 120 PARCL^WT^ proteins (cyan) and 120 miR399 (red) present. There are miRNAs within and at the interface of the condensates, but also isolated throughout the bulk solvent, which could suggest a limit to the amount of miR399 that can be recruited by droplets of these sizes.

Ostendorp et al.^37^ mutated five serines in the C-terminal region to alanine residues and reported that phase separation still occurred, but RNA binding was reduced. This led us to postulate that phase separation of the PLD and RNA binding of the C-terminal region function independently and can be manipulated individually. To investigate this prediction *in silico*, we repeated the PARCL simulations with these five serine residues mutated into glutamic acid. Figure 7A shows phase separation is unperturbed compared to wild-type, whereas the RNA binding is reduced.

We characterized each system’s interactions by calculating how often the different types of molecules came within contact distance (Table 1). Both the PARCL^WT^ and PARCL^C-term S−E^ proteins interacted with the miRNA, but the mutant tended to interact more with other PARCL proteins and less with the miR399 molecules than the PARCL^WT^. Figures 7C–7F show the disruptive effects the presence of RNA has on the phase separation at these concentrations, which matches the smaller droplets seen in experiments (Figure 7B). These results suggest that miRNA binding is likely independent from the regions of PARCL that drive phase separation, consistent with the experimental data.

**Table 1:** The C-terminal mutant S-E increases PARCL-PARCL interactions but reduces interactions between PARCL and miR399

System composition	Average interactions per frame of trajectory		
	PARCL-PARCL	PARCL-miR399	miR399-miR399
120 wild-type PARCL^WT^, 12 miR399	776.23	10.35	0.00
120 wild-type PARCL^WT^, 120 miR399	559.36	65.57	0.02
120 PARCL^C-term S−E^ mutant, 12 miR399	935.97	3.84	0.00
120 PARCL^C-term S−E^ mutant, 120 miR399	883.86	33.61	0.01

**Figure 8:**
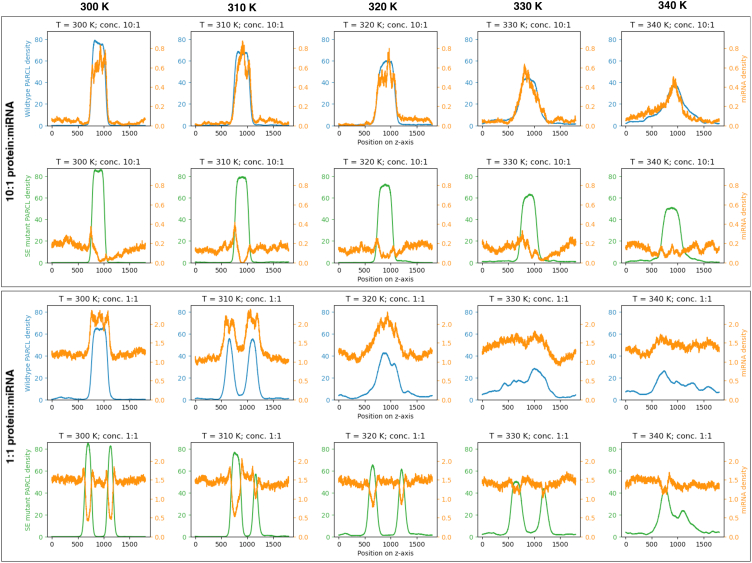
Mutations that reduce RNA binding affect the in-silico recruitment of miRNA into PARCL condensates We produced slab simulations across a range of temperatures and plotted the mean density profiles of the wildtype PARCL (blue), PARCL ^C−term S−E^ mutant (green), and miRNA (orange) across the last 100 ns of a 200 ns simulation. Density is calculated as beads per 1 Å section along the z axis. Columns indicate the temperature of the simulation, from 300 K (left) to 340 K (right). In the top two rows, the simulations contain 120 protein molecules and 12 miR399 molecules. In the bottom two rows, the simulations contain 120 protein molecules and 120 miR399 molecules. The density of the PARCL proteins is marked according to the left y axis, while the miRNA density is plotted against the right y axis. Wildtype PARCL has a stronger overlap with the miRNA density, indicating that the miR399 is recruited into the condensates. In contrast, the miRNA density is higher outside of the mutant PARCL droplets. The mutant also displays higher dense phase concentration than the wild-type, and maintains a sharp phase interphase at higher temperatures than the wildtype PARCL.

We also attempted to produce simulations to form phase diagrams for each component, at both 1:1 and 10:1 concentrations of protein:miRNA. We found that the slabs would often split into two droplets, which we believe is due to the starting configurations of the molecules. While this prevents a full phase diagram, we have plotted the density profiles of the last 100 ns of the 200 ns simulations (Figure 8). The wild-type PARCL and miRNA density profiles peak in the same regions of each simulation. In contrast, the minima of the miRNA density profiles are frequently contained at the peaks of the PARCL^C-term S−E^ mutant’s density profiles. The PARCL mutant displayed higher dense phase concentration across temperatures than the wild-type, while the wild-type lost its slab or droplet structure at lower temperatures than the mutant. For both PARCL variants, raising the amount of miRNA resulted in more miRNA in the bulk solvent relative to the slab, indicating a limit to the amount of miRNA that a PARCL condensate can recruit.

In summary, our analysis has shown that coarse-grained MD simulations coupled with an analysis of contact dynamics can be used as an effective tool to make predictions about PARCL phase separation behavior.

## Discussion

LLPS has recently been identified as an important mechanism in biology for compartmentalizing cells. This process leads to the formation of biomolecular condensates that have been implicated in stress granules, transcription machinery, and protein degradation. Our understanding of the nature of condensate formation has advanced significantly through experimental and computational approaches, including the development of powerful molecular dynamics frameworks to study phase separation *in silico.* Here, we applied a coarse-grained MD model to guide predictive protein engineering approaches. We studied the plant protein PARCL that has been shown to exhibit condensate formation *in vivo* and *in vitro*. We use this protein as a case study for evaluating the agreement between our framework of computational predictions and experiments (Figure 1), and to explore the predictive power for the rational design of targeted mutations.

We performed coarse-grained MD simulations of the phloem protein PARCL using the hydrophobicity scale model proposed in Regy et al.*,*^41^ allowing us to simulate, visualize, and quantify the protein’s phase separation behavior (Figure 2). Phase separation of PARCL can be observed experimentally *in vitro* and *in vivo* using fluorescent tagging using eYFP.^37^ To evaluate the impact of eYFP, we conducted a series of control MD simulations and experiments. We find that the eYFP domain does not drive LLPS as it fails to form condensates alone *in silico* (Figure 2A), *in vitro* (Figure 2D), or *in vivo* (Figure 2F). We produced simulations using an eYFP-PARCL construct (Figures S1–S3), but although this captured the formation of liquid clusters (Figure S1), the eYFP domain resulted in smaller clusters (Figure S2). Larger, slab-like droplets, as seen in our PARCL simulations, could not be maintained and therefore phase diagrams could not be produced to characterize eYFP-PARCL phase separation. Given that our experimental results show eYFP-PARCL forming large condensates, we believe that this is a limitation of the model and parameters used. Recent improvements including the CALVADOS 3^45^ and Mpipi-recharged^46^ models that more accurately capture interactions involving globular domains will be investigated in future work.

From a simple, distance-based analysis of which amino acids came within contact distance during the simulations, we identified candidate residues that may drive phase separation: a web of interactions centered around tyrosines (Figures 3 and S3–S5) in the PLD. While all the tyrosines within the PARCL sequence were involved in this network, this analysis highlighted which tyrosine residues had higher or lower involvement. We designed a mutant, PARCL^PLD Y−E^, in which the 6 tyrosines with the highest contact frequency were changed to glutamic acid. We confirmed that this mutant is less prone to phase separation *in silico*, *in vitro* and *in vivo* (Figure 4). In further simulations, we varied which of the tyrosines were mutated to glutamic acid, providing a wider scale of mutation testing *in silico* than would be practical in experiments. This showed how mutating different amounts of the tyrosines affects the phase separation behavior (Figure 5). Comparing the strength of the effect showed that while reducing the number of tyrosines generally reduced phase separation, the choice of which residues to mutate is more important. We produced slab simulations across a range of temperatures and plotted the HPS energy value against the mutants’ temperature-concentration relationships. For each quantity of mutated residues, there was a range of HPS energy scores. In some cases, this followed the expectations established by the PARCL^WT^ simulations. In other cases, this differs from expectations, such as the four least contacted tyrosine residues; a random selection of four tyrosines had less effect on LLPS as indicated by the HPS energy. Our simulations showed that the PARCL^PLD Y−E^ mutant was more disruptive to LLPS than any other tested mutant with 6 or fewer mutated residues. The simulations also demonstrated that phase separation behavior cannot be fully predicted from a linear combination of the contribution of individual amino acids, but that the HPS-Urry model can simulate the effects of mutations. Developing a more sophisticated method of learning the effects of potential mutations or proposing mutations for a desired effect without performing exhaustive combinations of simulations would be an interesting strand to follow and is something we are exploring.

We included both miRNA and PARCL in a simulation and found that, even though the simulation did not accurately capture miRNA structure, it identified a potential nucleic acid binding region in PARCL (Figure 6). We simulated a mutant, PARCL^C-term S−E^, in which 6 serine residues near the C-terminus were mutated to glutamic acid. These residues showed low contact propensity in wild-type simulations, and the mutants were able to phase separate with and without the presence of miRNA (Figures 7 and 8). The simulated mutants made less contact with miRNA than the wildtype, recapitulating experimental findings that phase separation and RNA binding can be independently targeted in PARCL.^37^

This study has demonstrated the effectiveness of coarse-grained MD simulations and contact analysis in guiding mutational studies aimed at perturbing condensate formation and protein-RNA interaction. Moreover, all the *in-silico* predictions could be experimentally validated *in vitro* and even *in vivo*. We believe the workflow presented here may be of general applicability for targeted engineering of condensate formation and protein-RNA interactions.

### Limitations of the study

We used coarse-grained molecular dynamics simulations in this study. Such models are approximations and depend on the chosen force fields. While the results were broadly in agreement, we noted a change in the ranking of specific pairs of residues for different parameter sets. We point out that with the explored parameter sets, we failed to accurately reproduce phase separation of eYFP-PARCL constructs. Also, the *in vitro* experiments contain crowding agents that we did not include in the model. The comparisons between the simulations and the *in vivo* results are thus limited by these approximations.

## Resource availability

### Lead contact

The lead contact, Ruth Veevers, can be reached at ruth.veevers@jic.ac.uk.

### Materials availability

The study did not generate any unique reagents.

### Data and code availability

• Data: We use very similar input files for each experiment listed, and provide the input files for the wildtype PARCL simulations as an example at https://github.com/ruth-veevers/Veevers2025SupportingCode. All raw data are available on request from the lead contact.
• Code: The simulations were produced using GENESIS MD, which is publicly available software. Code developed for this study to produce contact counts from trajectories is available at https://github.com/ruth-veevers/Veevers2025SupportingCode.
• All other items: Any additional information required to reanalyze the data reported in this paper is available from the lead contact upon request.

## Acknowledgments

This article is part of a project that has received funding from the European Research Council (ERC) under the European Union’s Horizon 2020 research and innovation program (Grant agreement No. 810131), and supported by the Deutsche Forschungsgemeinschaft (KE 856/8-1 to J.K.). J.K. acknowledges financial support to purchase the MALDI-TOF/TOF by the Deutsche Forschungsgemeinschaft (Grant agreement No. 469113358). We wish to express our thanks to three anonymous reviewers for their detailed and constructive feedback and excellent suggestions.

## Author contributions

R.V.: Conceptualization, formal analysis, investigation, methodology, visualization, writing – original draft, writing – review and editing; S.O.: Conceptualization, protein purification, resources, writing – review and editing; A.O.: Conceptualization, *in vitro*/*in vivo* phase separation assays, resources, writing – review and editing; J.K.: Conceptualization, funding acquisition, methodology, supervision, writing – review and editing; R.J.M.: Conceptualization, funding acquisition, methodology, supervision, writing – review and editing.

## Declaration of interests

The authors declare that they have no conflict of interest.

## STAR★Methods

### Key resources table

**Table:** 

REAGENT or RESOURCE	SOURCE	IDENTIFIER
**Antibodies**		

antiGFP-Nanobody	Katoh et al.^47^	Addgene plasmid #61838; RRID:Addgene_61838

**Bacterial and virus strains**		

Agrobacterium tumefaciens strain: LBA4404	GoldBio	Cat#CC-107-10x50
Escherichia coli BL21 strain: Rosetta 2 (DE3)	Merck	Cat#71400-4

**Chemicals, peptides, and recombinant proteins**		

Polyethylene glycol 3350	Sigma-Aldrich	Cat#P4338
cOmplete protease inhibitor cocktail tablet	Roche	Cat#4693132001
RNase A	Applichem	Cat#A2760,1000
DNase I	AppliChem	Cat#A3778,0500
Lysozyme	Applichem	Cat# A3711,0050
Thrombin protease	Cytiva	Cat#27084601

**Experimental models: Organisms/strains**		

*Nicotiana benthamiana*	N/A	N/A

**Recombinant DNA**		

pET28a-eYFP-AtPARCL wildtype	Ostendorp^37^	N/A
pET28a-eYFP-AtPARCL ctermS-E	Ostendorp^37^	N/A
pET28a-eYFP-AtPARCL PLD	This study	N/A
pET28a-eYFP	This study	N/A
pGEX6P1-GFP-Nanobody	Katoh et al.^47^	Addgene plasmid #61838
pEG104-eYFP-AtPARCL wildtype	Ostendorp^37^	N/A
pEG104-eYFP-AtPARCL cterm S-E	Ostendorp^37^	N/A
pEG104-eYFP-AtPARCL PLD	This study	N/A
pEG104-eYFP	Thus study	N/A

**Software and algorithms**		

GENESIS	Jung et al.^36^	https://github.com/genesis-release-r-ccs/genesis-2.1.0beta_cgdyn?tab=readme-ov-file
GENESIS input files	This study	https://github.com/ruth-veevers/Veevers2025SupportingCode
VMD	Humphrey et al.^48^	https://www.ks.uiuc.edu/Research/vmd/
MDAnalysis	Gowers et al.^49^; Michaud-Agrawal^50^	https://www.mdanalysis.org/
MD contact counting code	This study	https://github.com/ruth-veevers/Veevers2025SupportingCode

**Other**		

ÄKTAprime plus	Cytiva	Cat#11001313
Keyence BZ-X810	Keyence	https://www.keyence.com/products/microscope/fluorescence-microscope/bz-x700/models/bz-x810/
Ultraflex Xtreme MALDI-TOF/TOF	Bruker	https://www.bruker.com/en/products-and-solutions/mass-spectrometry/maldi-tof/ultraflextreme.html
HisTrap FF 5mL column	Cytiva	Cat#17525501
Branson Sonifier 250	FisherScientific	Cat#10588013
HiLoad 16/600 Superdex200 pg	Cytiva	Cat#28989335
Vivaspin 20 MWCO: 10000 Da	Sartorius	Cat#VS2001
Spectra/POR 7 MWCO 10000 Da	Carl Roth	Cat# E871.1
Glutathione high capacity magnetic agarose beads	Millipore	Cat#G0924

### Experimental model and study participant details

#### Protein purification

EYFP-tagged and untagged PARCL proteins were produced and purified as described previously^37^ and set to 200 μM as stock concentration. In brief, proteins were expressed in *E.coli* BL21 Rosetta2 (DE3) cells in 400 mL autoinduction medium (Studier 2005). The cultures were harvested by centrifugation at 5000 x g and lysis was carried out in 60 mL of lysis buffer (50 mM Tris-HCl pH 7.5, 300 mM NaCl, 10 mM imidazole, 1 mM AEBSF, 1 mM DTT, 1x cOmplete protease inhibitor tablet) supplemented with 1 mg/mL lysozyme, 0.1 mg/mL DNase I and 0.1 mg/mL RNAse A. After sonication of the resuspension on ice (Branson Sonifier 250, 8 times 30 s, duty cycle 50%, output 6), the lysate was centrifuged at 35 000 x g at 12°C. Purification was achieved in two consecutive steps using nickel affinity chromatography followed by size exclusion chromatography. The centrifuged lysate was loaded onto a 5 mL HisTrap FF column connected to an ÄKTA prime plus FPLC system run at 1 mL/min. The loaded column was washed with lysis buffer containing 1 M NaCl and proteins were eluted using an imidazole gradient from 0 to 1 M in buffer B (50 mM Tris-HCl pH 7.5, 300 mM NaCl, 1 M imidazole) over 15 column volumes. Fractions containing desired proteins were pooled and dialyzed overnight in 2 L of dialysis buffer (50 mM Tris-HCl pH 7.5, 200 mM NaCl, 1 mM DTT) in a SpectraPor dialysis membrane (MWCO: 10000 Da). Thrombin (20 u) was added to remove the 6xHis tag. On the next day, dialyzed proteins were subjected to size exclusion chromatography using a HiLoad 16/600 Superdex 200 pg column equilibrated in gel filtration buffer (25 mM Tris-HCl pH 7.5, 200 mM NaCl, 1 mM DTT). The quality of all purified proteins was further accessed by MALDI-TOF mass spectrometric measurements using a Bruker Ultraflex extreme MALDI-TOF/TOF mass spectrometer.

#### *In vitro* phase separation assays

To induce protein condensation, purified proteins were diluted to a final concentration of 10 μM with 1x condensation buffer (50 mM Tris-HCl pH 7.5, 150 mM NaCl). Subsequently, 10% (w/v) PEG3350 was added as a crowding agent and incubated for 2 min prior to the measurements. 5 μL were transferred onto a glass slide (Labsolute, Th. Geyer GmbH, Germany) and carefully covered with a siliconised circular cover slide (22 mm diameter, Jena Bioscience, Germany). Condensation formation was observed by fluorescence microscopy using a BZ-X810 fluorescence microscope (Keyence, Germany) using a 100× oil immersion objective equipped with an eYFP filter system (Chroma, USA). All microscopic analyses were performed at an exposure time of 17 ms as a single z-plane without any black balance.

#### Sampling eYFP accessibility in PARCL condensates

To test if eYFP is solvent-exposed and thus accessible within PARCL condensates, studies with an anti-YFP-nanobody (Addgene plasmid #61838)^47^ were carried out. 15 μL glutathione high capacity magnetic agarose beads slurry (Millipore, Germany) were transferred to 1.5 mL reaction tubes and washed 3 times with 200 μL condensation buffer. Beads were coupled by incubating with 100 μM anti-GFP-nanobodies in condensation buffer for 30 min at RT with shaking at 150 rpm. Free nanobodies were removed by three wash steps with 200 μL condensation buffer. Then 20 μM eYFP or wildtype eYFP-AtPARCL in condensation buffer, which was supplemented with 10% PEG3350 and incubated for 20 min, was directly transferred to the beads. After 30 min of incubation, samples were transferred onto a microscope glass slide, covered with a coverslip and observed by fluorescence microscopy using the Keyence BZ-X810 fluorescence microscope equipped with an eYFP filter.

#### *In vivo* condensation studies

PARCL mutants were cloned into pEarlyGate104 (pEG104) achieving N-terminally eYFP-tagged PARCL proteins expressed under the strong 35S promoter. The plasmids were transformed into agrobacterium tumefaciens cells (LBA4404) and were cultivated on solid LB medium (supplemented with 100 μg/mL Kanamycin) at 28°C overnight. Bacterial colonies were resuspended in infiltration medium (1/4 murashige and skoog medium, 100 μM acetosyringone, 1% sucrose, 0.005% Silwet 77 pH 6.0) and diluted to an OD600 of 0.5. These suspensions were infiltrated into 6 week old tobacco plant leaves. After cultivation of the plants over night at 25°C in dark, plants were cultivated at 25°C under low light conditions for further two to three days prior to sampling. Infiltrated areas were cut out, mounted onto a glass slide and covered with a glass coverslip. Cells were microscopically analyzed using a Keyence BZ-X810 equipped with an eYFP filter and its companion analysis software.

### Method details

#### Framework

The initial simulation set-up differs based on the type of the target sequence. For proteins, 3D coordinates for the tertiary structure are predicted using AlphaFold2^51^ and regions of disorder are predicted with IUPred3.^52^ The IUPred3 output and the AlphaFold2 confidence scores are used to establish which parts of the structure should be treated as folded and which parts should be treated as IDRs. From the predicted structure and predicted list of IDRs, a coarse-grained representation of the protein is generated using GENESIS.^36^ Other molecule types are all treated as fully intrinsically disordered, and CG representations are generated directly from the sequence as a line of beads which is relaxed with a short period of simulation. A further GENESIS tool is used to duplicate the molecules into a grid, and two GENESIS simulations are run. Each establish a “unit cell” surrounding the grid of molecules and imposing periodic boundary conditions such that if a bead leaves the cell boundaries, it re-enters with the same trajectory from the opposite boundary. This simulates a system of infinite size containing infinite repetitions of the contents of the central unit cell. In the first simulation the bounds of this unit cell are placed around the grid of molecules and then gradually shrunk until the unit cell reaches a uniform target size. The second, longer simulation takes the output of the shrinking simulation and runs for 100 nanoseconds to capture phase behavior. The simulations were checked for energy convergence and extended for the same duration until convergence. The dynamic coordinates at intervals along the simulated trajectory are then analyzed to find distances between pairs of atoms, taking note of pairs of residues that come within the sum of their radii. These are used to identify attraction hubs to mutate in further simulations and in *in vitro* experiments.

#### Preprocessing and set-up of initial structures

As proteins can have both structured and disordered regions, we use two external tools to derive structural information. IUPred3^52^ is strictly a predictor of disorder, which we run in its short disorder mode with medium smoothing to obtain a list of regions within the sequence that are predicted to be disordered. AlphaFold2^51^ provides a prediction of the structure of ordered regions and assigns a confidence score to its prediction for each residue. Residues assigned low confidence by AlphaFold2 are added to the regions of predicted disorder for two reasons: it has been shown to be correlated with disorder^53^^,^^54^^,^^55^ and it prevents over-reliance on structures that are likely to be incorrect.

#### Building and running CG MD simulations

The following steps are carried out within GENESIS.^36^ We use a coarse-grained slab simulation for phase separation as described in previous work.^24^^,^^26^^,^^27^

We create a coarse-grained representation of the predicted structure which uses one “bead” particle to represent each residue, centered on its C_alpha_ atom. Input files created alongside this representation specify parameters such as native contacts and angles. To these files, we add markers denoting the locations of predicted IDRs.

We duplicate this representation of the molecule, creating identical copies along the x, y, z axes to create a 2 × 2 × 30 grid of molecules. The dimensions of this system will vary, and so to compare behavior at similar concentrations we perform a shrinking step to unify the final volume. This step is a short simulation using the periodic boundary condition in which we reduce the unit cell dimensions to 180 Å by 180 Å by 1800 Å. This results in a protein concentration of 3.4 mM which is much higher than the 10 μM used *in vitro*, so we do not add a crowding agent.

For the second simulation, we use the periodic boundary condition with a fixed unit cell of 180 Å by 180 Å by 1800 Å and an implicit solvent with an ionic strength of 0.15 M. The volume and the temperature, 300 K, are held constant throughout the simulation. The hydrophobicity scale parameters for amino acids^27^ and nucleic acids^41^ are taken from previous studies. We run each simulation for 10,000,000 steps, with each step corresponding to 0.01 picoseconds. The coordinates of all beads in the system are written out to a coordinate file every 10,000 steps, which results in a 1,000-frame trajectory. Simulations are checked for convergence and extended where necessary.

### Quantification and statistical analysis

#### Phase diagrams

We have probed the temperature-concentration relationships within these simulations by performing simulations for the construction of phase diagrams. These use inbuilt GENESIS shrinking effects to further compress the systems to a smaller 180 Å cube, then remove this effect and continue the simulation with a 180 Å by 180 Å by 1800 Å unit cell. The simulations are performed across a range of temperatures. In their analysis, the density is calculated in units of beads per 1 Å slice of the z axis. We note where the protein does not maintain a slab, and where it does maintain a clear slab we take the mean density for the condensed phase from within its center. The mean density for the solute phase is taken outside the slab.

#### Dynamic analysis of LLPS simulations

We simulate at a coarse, residue-level resolution so it is not possible to assess exact interatomic interactions. Instead, we focus on residues that pass one another within a close enough distance to potentially make contact. We consider two residues to be in potential contact if the distance between them is less than the sum of their radii calculated from estimated residue volumes.^56^ We calculated inter-residue distances using MDAnalysis.^49^^,^^50^

Trajectory images are rendered in VMD^48^ using Tachyon ray-tracing.
