# Supplementary material for: Controlled liquid-liquid phase separation via the simulation-guided, targeted engineering of the RNA-binding protein PARCL
Source: iScience. 2025 Jun 11;28(7):112852. doi: 10.1016/j.isci.2025.112852 (PMC12256297; doi:10.1016/j.isci.2025.112852)
Supplement: Document S1. Figures S1–S6 [file mmc1.pdf]

iScience, Volume *28*

Supplemental information

Controlled liquid-liquid phase separation via the simulation-guided, targeted engineering of the RNA-binding protein PARCL

Ruth Veevers, Steffen Ostendorp, Anna Ostendorp, Julia Kehr, and Richard J. Morris

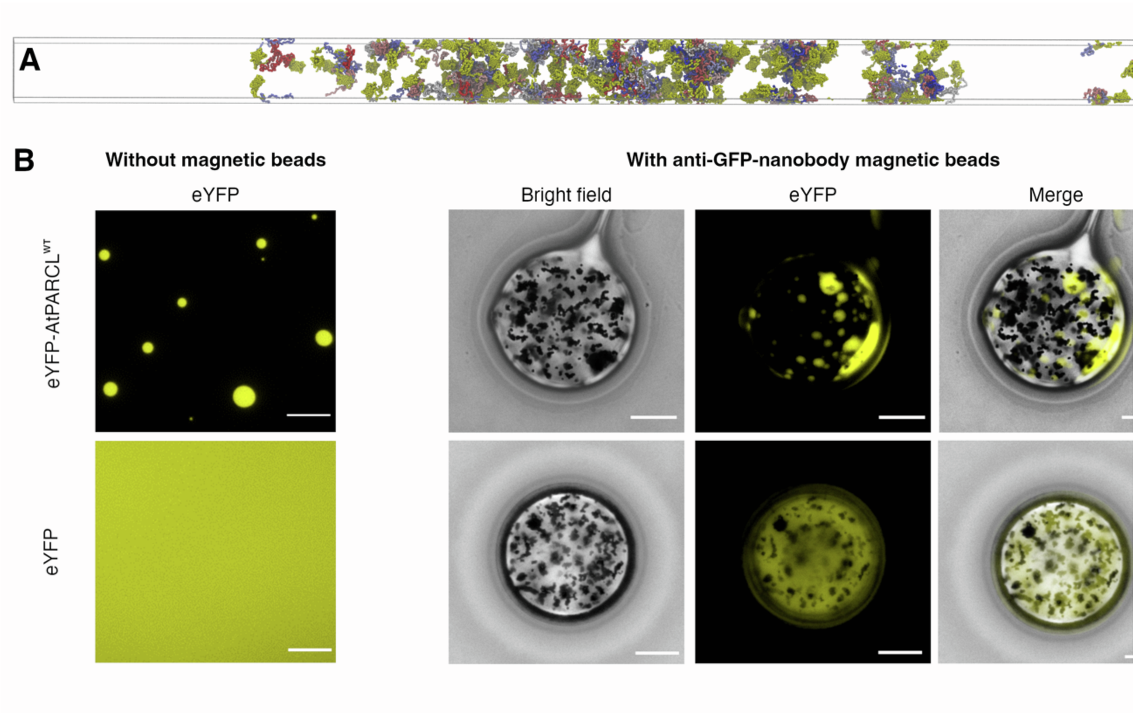

Figure S1 – Experiments suggest that eYFP-PARCL forms biocondensates that have eYPF accessible from the surface. A Rendering of the coordinates of 120 eYFP-PARCL proteins after 100 nanoseconds of coarse-grained MD simulation. The periodic boundary was extended to 3600 Å due to the increased size of the molecules. The eYFP domains are coloured yellow and treated as structured; each molecule's PARCL domain is assigned a unique shade of the blue-white-red spectrum and treated as intrinsically disordered. Liquid cluster formation is indicative of phase separation, however, the simulations failed to maintain a slab structure, hindering the determination of a phase diagram. Despite the computational results being inconclusive in term of phase separation, the eYFP domains tend to reside at the cluster surfaces which is consistent with the experimental observations. Rendered in VMD using the Tachyon ray-tracer. **B** Testing eYFP accessibility in eYFP-PARCL condensates *in vitro* using magnetic beads coupled to anti-GFP-nanobodies. 20 μM protein was used and condensation was induced by adding 10 % PEG3350 in 1x condensation buffer. In contrast to free eYFP not undergoing LLPS, which shows uniform binding to the magnetic beads, eYFP-tagged PARCL condensates can be isolated on the beads, supporting the simulation results of solvent exposed eYFP in the eYFP-PARCL condensates. Scale bar: 10 μm. Related to Figure 2.

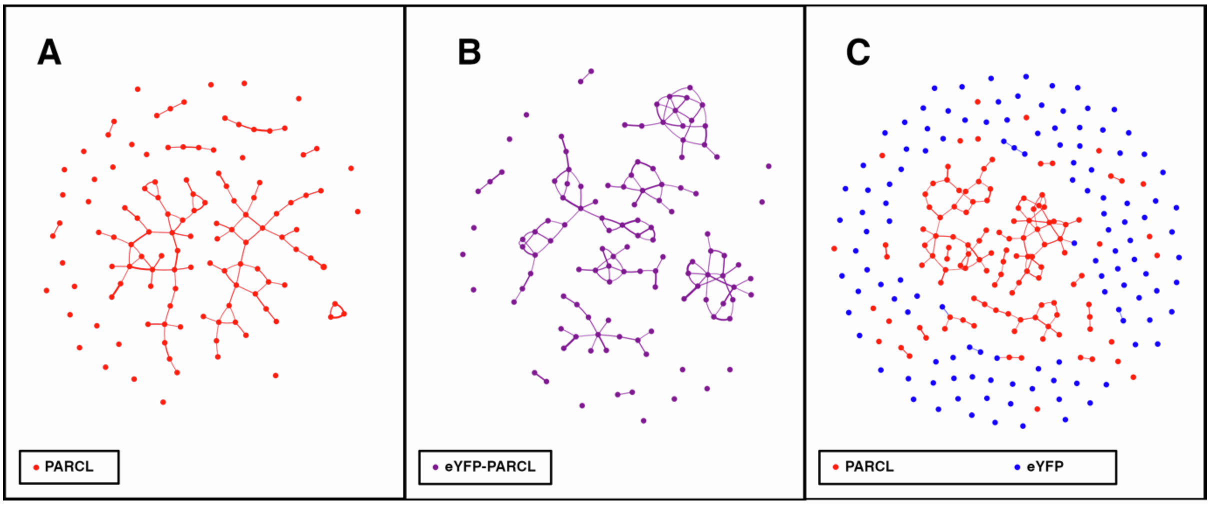

Figure S2: The eYFP domain results in more small droplets of eYFP-PARCL, and does not phase separate alone. Network representations of the simulations, using trajectory coordinates from one frame after 100 ns of simulation. Plots are an abstracted representation of the system in order to demonstrate connected proteins. Each node represents one protein. An edge is drawn between two nodes if the proteins they represent have at least 5 pairs of residues making contact, representing an interaction of at least 4 kT in strength. Edge thickness corresponds to the number of contacting residue pairs above 5. Networks were created and plotted using the python library PyVis, which arranged the node layout for readability; node position does not correspond to protein coordinates within the simulation. The networks are taken from the following simulations: A: The wildtype PARCL (red nodes) simulation shown in the droplet snapshot in Fig 2A; B: the eYFP-PARCL construct (purple nodes) simulation; C: the simulation free eYFP (blue nodes) and PARCL (red nodes) shown in Figure 2C. Related to Figure 2.

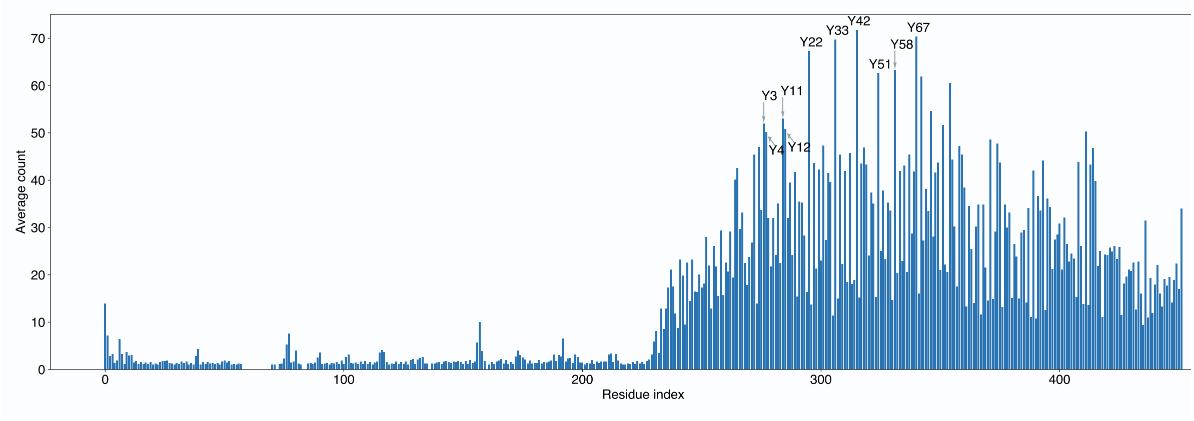

Figure S3: eYFP-PARCL residue mean contact counts. The first 274 residues belong to the eYFP domain. Tyrosine residues have been labelled according to their position in the PARCL sequence. While the eYFP-PARCL simulations did not accurately capture system-wide phase behaviour seen in experiments, we investigated residue-level behaviour. We found that despite the inclusion of the eYFP domain, the general ranking of the PARCL residues by contact frequency remained the same, apart from a reduced role for the initial methionine which was hindered by its adjacency to the eYFP structure. The residues of eYFP themselves

made only minimal contact to other molecules. The PLD tyrosine residues that were identified as important in Figure 3 were again the most contacted residues in the eYFP-PARCL simulations. Related to Figure 3.

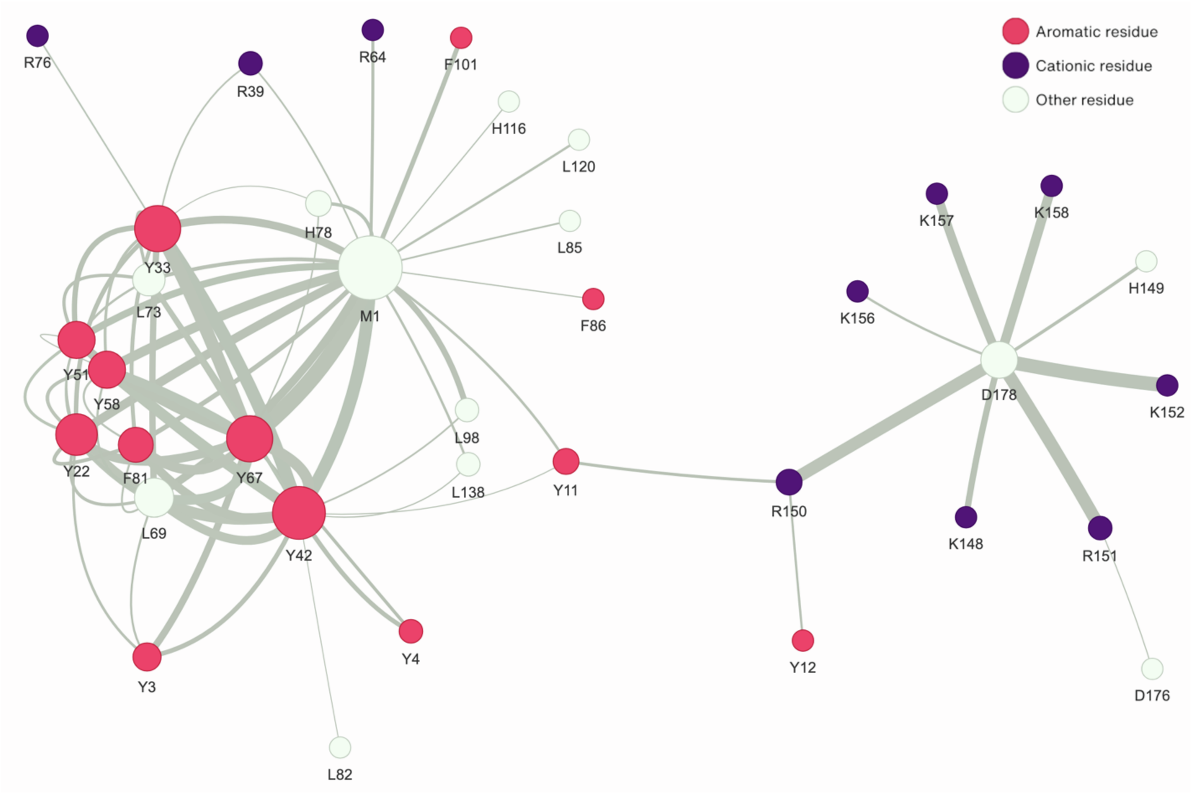

Figure S4: Residue pairs that most frequently interact in the final 100ns of the PARCL wild-type simulation. Node size represents number of contacts between the residue and any other residue. Edge width represents number of contacts between the two connected nodes. Only pairs that were counted at least 350 times are plotted for readability. Related to Figure 3.

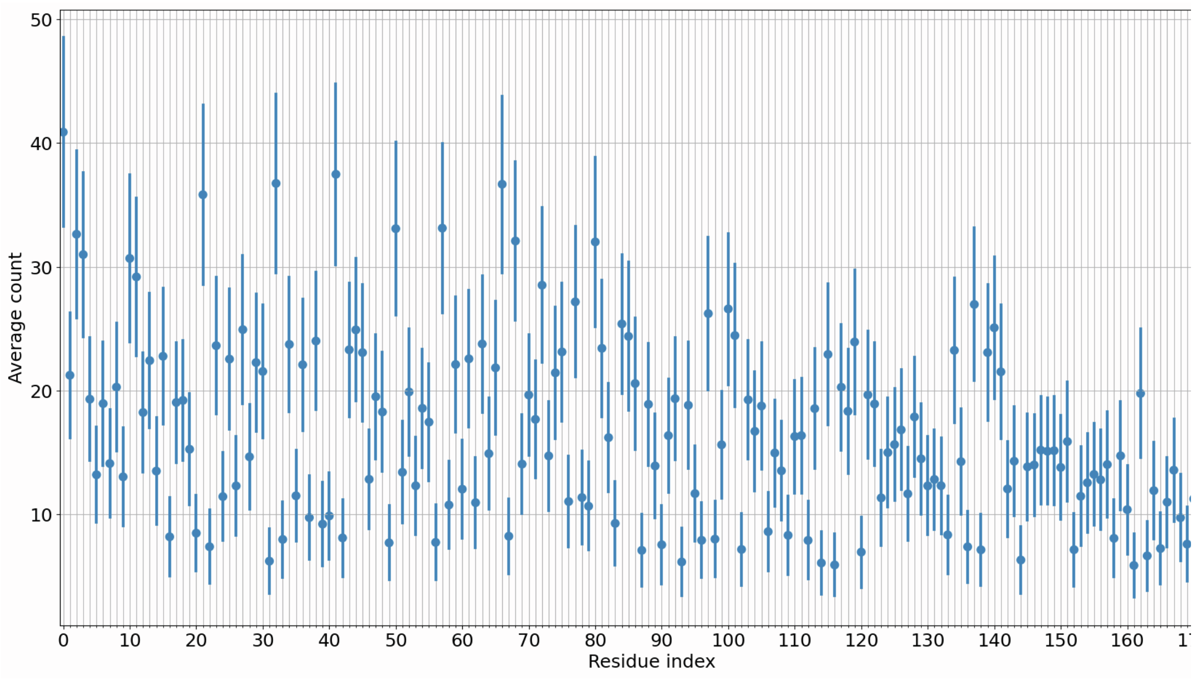

Figure S5: Version of Fig 3 that shows error bars. Points represent the mean; lines indicate standard deviation. Contact counts are calculated for each frame in the final 100ns of the simulation of wildtype PARCL. Related to Figure 3.

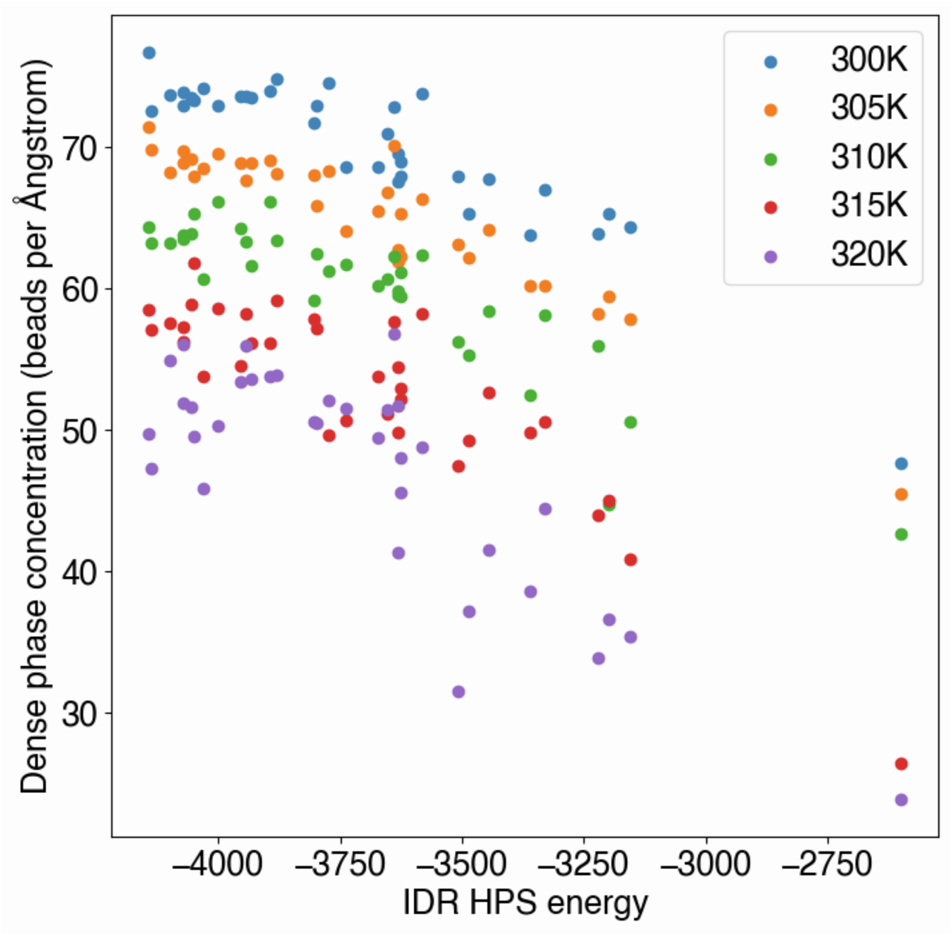

Figure S6: Correlation between IDR HPS energy at 300K and dense phase concentration at 5 different temperatures between 300K and 320K. We produced 200ns slab simulations at 300K, 305K, 310K, 315K, and 320K for each potential PARCL mutant and calculated the mean concentration (in terms of residues per 1 Å section of the *z*-axis) of the slab for the final 50ns, plotted against the mean IDR HPS energy contribution for the same period. Related to Figure 5.
